# Duration‐Dependent Effects of Rivaroxaban on Inflammation and Valve Calcification in Aortic Stenosis: Clinical and In Vitro Insights

**DOI:** 10.1111/jcmm.70927

**Published:** 2025-10-31

**Authors:** Magdalena Kopytek, Marcin Zuwała, Radosław Chudy, Weronika Włóczyk, Michał Ząbczyk, Jacek Piątek, Anetta Undas, Joanna Natorska

**Affiliations:** ^1^ Department of Thromboembolic Disorders Institute of Cardiology, Jagiellonian University Medical College Krakow Poland; ^2^ Krakow Centre for Medical Research and Technologies St. John Paul II Hospital Krakow Poland; ^3^ Students' Scientific Group of Thromboembolic Disorders Jagiellonian University Medical College Krakow Poland; ^4^ Department of Anesthesiology and Intensive Care Medicine St. John Paul II Hospital Krakow Poland; ^5^ St. John Paul II Hospital Kraków Poland

**Keywords:** bone morphogenetic protein‐4, direct oral anticoagulants, inflammation, nuclear factor‐κB, osteocalcin, valve interstitial cells

## Abstract

Direct oral anticoagulants (DOACs) inhibited calcification and extracellular matrix remodelling in valve interstitial cells (VICs). We assessed whether DOACs affect inflammation and calcification both in vitro and in stenotic aortic valves from patients with severe aortic stenosis (AS). We enrolled 52 patients with AS, including 22 with concomitant atrial fibrillation taking DOACs. Stenotic leaflets obtained during surgery were assessed for osteocalcin and bone morphogenetic protein 4 (BMP‐4) by immunostaining. Serum interleukin‐6 (IL‐6), transforming growth factor‐β (TGF‐β) and matrix metalloproteinase‐9 (MMP‐9) were measured by ELISA. In vitro, VICs were treated with rivaroxaban (1 or 10 μg/mL) to evaluate BMP‐4 and nuclear factor kappa B (NF‐κB) expression via immunofluorescence. A median of DOAC therapy was 29.5 [Q1‐Q3 20–48] months. The percentage of valvular osteocalcin, but not BMP‐4 positive areas, was 39.6% lower in patients taking DOACs compared to the remainder (16.3% ± 4.8% vs. 27% ± 6.3%, *p* < 0.0001). Importantly, both valvular BMP‐4 and osteocalcin expression correlated inversely with the duration of DOAC therapy (*r* = −0.92 and *r* = −0.78, both *p* < 0.0001). Additionally, DOAC‐treated patients had lower serum MMP‐9 (−58.1%, *p* < 0.0001) and TGF‐β (−7.9%, *p* = 0.026) but not IL‐6 (*p* = 0.092) compared to the non‐DOAC group. DOAC duration correlated with MMP‐9 (*r* = −0.89, *p* < 0.0001) and IL‐6 (*r* = −0.63, *p* = 0.0015) levels. In vitro, rivaroxaban reduced NF‐κB and BMP‐4 expression in VICs in a time‐dependent manner (all *p* < 0.01), regardless of dose. We showed that DOACs, in a time‐dependent manner, exert anti‐inflammatory and anti‐calcific effects within aortic stenotic valves and in vitro in VICs, suggesting long‐term DOAC therapy benefits.

## 1 Introduction

Aortic stenosis (AS) is a progressive disease characterised by calcification and narrowing of the aortic valve area, resulting in significant hemodynamic changes and increased cardiovascular morbidity and mortality [1]. The cellular architecture of the aortic valve is defined primarily by two types of cells: valve endothelial cells (VECs), which line the outer surface and act as a protective barrier limiting lipid accumulation and inflammatory cell infiltration and valve interstitial cells (VICs), which reside within the fibrosa, spongiosa and ventricularis layers and represent the predominant cellular population of the valve leaflets [2]. The progression of AS is driven by complex interactions between lipids, inflammatory and coagulation factors, leading to valve calcification [3]. These changes in the cellular environment activate VICs, which are highly responsive to osteogenic mediators, such as transforming growth factor‐β (TGF‐β) and bone morphogenetic proteins (BMPs) [4, 5]. After transformation of VICs, they change their phenotype and are characterised by the expression of specific markers such as α‐smooth muscle actin for myofibroblasts (chondrogenic differentiation) and osteopontin, osteocalcin as well as BMP‐2 and ‐4 for osteoblast‐like fibroblasts (osteogenic differentiation) [5]. BMPs promote valve calcification by activating the Smad1/5/8 and Wnt/β‐catenin signalling pathways, resulting in the up‐regulation of the key osteoblast transcription factor—Runt‐related transcription factor 2/core‐binding factor α‐1 (Runx2/Cbfα1). Runx2/Cbfα1 enhances the expression of proteins directly associated with calcification and osteoblast differentiation such as osteopontin, bone sialoprotein and osteocalcin [4, 5]. A previous study has demonstrated that the valvular ossification and calcification is under the regulation of nuclear factor kappa B (NF‐κB), suggesting its important role in the pathophysiology of AS [6]. NF‐κB is activated by tumour necrosis factor α (TNF‐α) or TGF‐β [7, 8], secreted by monocytes/macrophages and causes an up‐regulation of interleukin 6 (IL‐6), which has been implicated in calcification of aortic valve leaflets in AS patients via BMP‐2 stimulation [9]. Moreover, the p65/c‐Rel heterodimer of NF‐κB has been shown to critically regulate expression of tissue factor (TF) [10], the valvular expression of which has been found in patients with severe AS in the area of fatty‐calcium deposits [11]. Interestingly, in vitro studies have demonstrated that inhibition of NF‐κB enhances osteoblast differentiation and bone formation by increasing alkaline phosphatase activity, Smad1/5/8 phosphorylation and mRNAs expression of osteoblast‐specific genes, such as type I collagen, osteopontin, osteocalcin and Runx2 [12, 13].

Direct oral anticoagulants (DOACs), including rivaroxaban, have emerged as a therapeutic option in various cardiovascular conditions. Recently published guidelines for the management of valvular heart disease recommended DOACs for stroke prevention in AS patients with atrial fibrillation [14]. However, their role in AS and the impact of long‐term DOAC therapy on disease progression remain largely unexplored. Recent data confirmed the anti‐calcification effect of rivaroxaban in VICs cultures as evidenced by reduced levels of both osteocalcin and osteopontin in conditioned media [15]. In addition, the authors have shown that rivaroxaban attenuates extracellular matrix remodelling reflected by decreased matrix metalloproteinase (MMP)‐1, ‐2 and ‐3 levels and inflammation proven by reduced levels of IL‐6, ‐32 and ‐34 [15].

This study aimed to evaluate the effects of rivaroxaban treatment on inflammatory markers, procoagulant factors and valve calcification in a cohort of AS patients. The study also investigated the dose‐dependent effects of rivaroxaban on the expression of inflammation and calcification markers in vitro in VICs.

## 2 Materials and Methods

### 2.1 Patients

A total of 52 patients were recruited for this study, including consecutive 22 patients with severe AS receiving DOACs due to concomitant atrial fibrillation and 30 patients with isolated severe AS not receiving DOACs. All participants underwent scheduled surgical aortic valve replacement at the Department of Cardiac Surgery and Transplantology at the St. John Paul II Hospital in Krakow between 2022 and 2023. Patients treated with DOACs were subjected to the bridging therapy, using enoxaparin 1 mg/kg twice daily. Bridging therapy duration lasted 48 h. Demographic data, medical history and therapeutic information were collected using a medical questionnaire. AS was diagnosed based on transthoracic echocardiography performed by an experienced cardiologist using a Toshiba APLIO 80 device (Toshiba, Tokyo, Japan). Severe AS was defined as an aortic valve area (AVA) < 1 cm^2^, and/or mean transvalvular pressure gradient (PG_mean_) ≥ 40 mmHg, and/or peak transvalvular velocity (*V*_max_) ≥ 4.0 m/s, according to the guidelines [14]. Hypercholesterolemia and arterial hypertension were diagnosed as previously described [16].

The following exclusion criteria were applied: atherosclerotic vascular disease requiring revascularisation, acute infection including infective endocarditis, rheumatic AS, diabetes mellitus, advanced chronic kidney disease, need for concomitant valvular surgery (e.g., mitral valve repair), percutaneous coronary intervention, recent (< 3 months) acute coronary syndrome or cerebrovascular episode, diagnosed malignancy and pregnancy. The valvular anatomy was confirmed intraoperatively, and patients with bicuspid valve and root/ascending aortic dilatation requiring intervention were excluded from the study. The diagnosis of atherosclerosis was based on angiographically documented coronary artery stenosis greater than 20% of the diameter, and such patients were excluded from the study to avoid any influence of nonobstructive atherosclerosis [17].

The Ethical Committee (Krakow Medical District Chamber, Poland, approval number: 8/KBL/OIL/2019) approved the study and all participants provided written informed consent in accordance with the Declaration of Helsinki.

### 2.2 Laboratory Investigations

Blood samples were collected on the day of surgery. Fasting venous blood was drawn from the antecubital vein between 7:00 and 9:00 AM in all AS patients before aortic valve replacement. Citrated blood (9:1, 0.106 M sodium citrate) was centrifuged at 2000 *g* for 20 min at 20°C, and EDTA or serum samples were centrifuged at 1600 *g* for 10 min at 4°C. All samples were aliquoted and stored at −80°C until analysis. Blood lipid profile (including total cholesterol, LDL, HDL and triglycerides), glucose, creatinine, CRP and fibrinogen levels were measured using standard laboratory techniques.

### 2.3 Aortic Valve Preparation

Aortic valve leaflets were collected during surgery and divided into three parts: one for histological and immunofluorescence staining, one for in vitro VIC isolation and culture and one frozen for future studies. Frozen leaflets were cryosectioned (4.5–5 μm) using a Leica CM1520 cryostat. Sections were mounted on Menzel‐Gläser SuperFrost Plus slides (Thermo Fisher Scientific, Braunschweig, Germany) and stored at −20°C for further morphological and immunofluorescence analysis.

### 2.4 VIC Culture Preparation

VICs cultures were acquired from non‐calcified fragments of stenotic aortic leaflets from patients with isolated AS and obtained as previously described [18]. The experiments were conducted on VICs between their third and fifth passages. When VICs reached 90%–95% confluence, they were subcultured into 6‐well plates at a concentration of 1 × 10^5^ cells per well in 2 mL of cell culture medium. VICs cultured in a standard medium (DMEM: low glucose medium, without L‐glutamine and with sodium pyruvate; Biowest, Nuaillé, France) served as a negative control. To initiate the process of calcification, VICs were maintained in an osteogenic medium containing CaCl_2_ (1.5 mmol/L, Chempur, Piekary Śląskie, Poland), β‐glycerophosphate disodium hydrate salt (10 mmol/L, Sigma‐Aldrich, St. Louis, MO, USA) and ascorbic acid (50 μg/mL, Chempur) for approximately 1 week prior to mechanistic experiments, allowing the cells to adhere, proliferate and establish a procalcifying microenvironment. The medium was changed twice weekly during this preconditioning period. To induce inflammation, VICs were cultured in the osteogenic medium supplemented with TNF‐α (hBA‐158: final concentration 50 ng/mL; Santa Cruz Biotechnology, Dallas, TX, USA) [18]. The impact of DOACs was investigated by treating the VICs with rivaroxaban (BAY 59–7939: Selleckchem, Houston, TX, USA) at concentrations of 1 and 10 μg/mL, corresponding to therapeutic doses. All VICs were cultured for 24 and 48 h. Each experiment was repeated three times using VICs isolated from randomly selected stenotic valves.

### 2.5 Valve Immunofluorescence Staining

The expression of calcification‐related factors, such as BMP‐4 and osteocalcin, was assessed in stenotic valves using the previously described procedure [19] and primary antibodies diluted at 1:100 for BMP‐4 and 1:200 for osteocalcin (both from Abcam, Cambridge, UK). The corresponding secondary goat antibody conjugated with AlexaFluor 488 (Abcam) was applied in the dark (1:1000). A negative IgG isotype control was performed routinely. An Olympus BX43 microscope (Tokyo, Japan) was used to visualise the images. The percentage of immunopositive areas in the valve sections was calculated relative to the total area of the analysed section [20]. Fluorescence intensity (FI) was measured based on green colour intensity in the RGB scale using ImageJ software (version 1.54). The program was also used to measure valve thickness.

### 2.6 VIC Immunofluorescence Staining

The expression of NF‐κB and BMP‐4 in VICs cultures was assessed using immunofluorescence as previously described [19], using the following primary antibody dilutions: NF‐κB at 1:500 and BMP‐4 at 1:100 in blocking serum (both antibodies from Abcam). The corresponding secondary goat antibody conjugated with AlexaFluor 488 (Abcam) was applied in the dark (1:1000). The number of immunopositive cells was counted across 300 consecutive cells per slide and 3 slides per each condition [19].

### 2.7 ELISA Testing

The concentrations of TGF‐β1, high‐sensitivity IL‐6 (hsIL‐6), TF and MMP‐9 (all from R&D Systems, Minneapolis, MN, USA) were quantified in the patients' plasma or serum samples in accordance with the manufacturers' instructions.

### 2.8 Statistical Analyses

All statistics were performed using the STATISTICA software (Version 13.3, TIBCO Software, Palo Alto, CA, USA). Categorical variables were presented as numbers and percentages, while continuous variables were presented as mean and standard deviation (SD) or median and quartiles Q1–Q3. Categorical variables were analysed by *χ* ^2^ with Yates' correction or two‐tailed Fisher's exact test. Normality was analysed by the Shapiro–Wilk test. Differences between the groups were compared using Student's *t*‐test or Mann–Whitney *U* test, as appropriate. Associations between variables were calculated using Spearman's correlation coefficients due to the small sample size (≤ 30 per group). To minimise the potential confounding effects of analysed factors, such as medications on AS severity, additional adjustments were performed using general linear models. For in vitro experiments, a dependent samples *t*‐test was used to compare the sample means from two related groups, and analysis of variance (ANOVA) was used to compare continuous variables between multiple groups. Post hoc comparisons were performed with the Tukey–Kramer HSD test. A *p*‐value < 0.05 was considered statistically significant.

## 3 Results

### 3.1 DOAC Treatment Duration and AS Severity

The study included 52 patients, of whom 22 (42.3%) were receiving DOAC therapy. This group consisted of patients on rivaroxaban (54.6%, 20 mg/day), apixaban (22.7%, 5 mg/day) and dabigatran (22.7%, 2 × 150 mg). A median duration of DOAC treatment was 29.5 [Q1–Q3 20–48] months. The remaining 30 patients (57.7%) had isolated AS and were not treated with DOACs. The baseline characteristics of the patients with AS are presented in Table 1. Patients in both groups did not differ in terms of demographic characteristics, risk factors and most medications, except for a higher frequency of aspirin use in the non‐DOAC group (Table 1). Similarly, there were no differences in baseline laboratory findings (Table 1). However, patients on DOACs were characterised by lowering *V*_max_ (−8.7%), PG_mean_ (−19.2%) and PG_max_ (−16.5%), but not AVA, compared to the others (Table 1). After adjustment for aspirin use, the difference in PG_mean_ and *V*_max_ remained significant (*p* = 0.04 and 0.044, respectively) but not for PG_max_ (*p* = 0.07). Moreover, patients treated with DOACs at least for 29 months compared to those treated with DOACs < 29 months had 11.4% lower PG_max_ and 4.5% lower *V*_max_, while we found no difference in PG_mean_ (Table 1). However, after adjustment for angiotensin‐converting enzyme (ACE) inhibitors, all 3 echocardiographic parameters differed between patients receiving long‐term and short‐term DOAC treatment (*p* = 0.002 for PG_max_, *p* = 0.002 for *V*_max_ and *p* = 0.047 for PG_mean_). We also observed 14.6% lower fibrinogen in patients taking DOACs ≥ 29 versus < 29 months (Table 1).

**TABLE 1:** Baseline characteristics of AS patients.

Variable	Isolated AS patients (*n* = 30)	AS patients on DOACs (*n* = 22)	*p*	DOAC therapy duration < 29 months (*n* = 10)	DOAC therapy duration ≥ 29 months (*n* = 12)	*p*
Age, years	68.6 ± 5.3	71.2 ± 6.4	0.11	72.0 [66.0–76.0]	73.0 [68.0–75.5]	0.72
Male, *n* (%)	17 (56.7)	15 (68.2)	0.58	7 (70)	8 (80)	0.62
BMI, kg m^−2^	30.6 ± 5.1	30.8 ± 5.0	0.88	31.2 [28.3–32.4]	33.2 [26.2–35.0]	0.58
**Risk factors, *n* (%)**						
Arterial hypertension	27 (90)	22 (100)	0.25	10 (100)	12 (100)	0.99
Hypercholesterolemia	28 (93.3)	20 (90.9)	0.99	9 (90)	11 (91.7)	0.99
Current smoking	7 (23.3)	1 (4.5)	0.12	1 (10)	0	0.45
**Medications, *n* (%)**						
Beta‐blockers	26 (86.7)	19 (86.4)	0.99	8 (80)	11 (91.7)	0.57
Acetylsalicylic acid	25 (83.3)	7 (31.8)	**0.0004**	2 (20)	5 (41.7)	0.38
ACE inhibitors	17 (56.7)	16 (72.7)	0.37	5 (50)	11 (91.7)	0.06
Statins	27 (90)	19 (86.4)	0.69	8 (80)	11 (91.7)	0.57
**Echocardiographic parameters**						
PG_mean_, mmHg	52 [44–70]	42 [37–49]	**0.0004**	45 [40–50]	40 [36–45]	0.09
PG_max_, mmHg	85 [73–103]	71 [67–78]	**0.009**	79 [74–82]	70 [66–71]	**0.003**
AVA, cm^2^	0.7 [0.6–0.8]	0.8 [0.7–0.9]	0.12	0.8 [0.7–0.9]	0.85 [0.7–0.9]	0.58
*V*_max_, m/s	4.6 [4.3–5.1]	4.2 [4.1–4.4]	**0.008**	4.4 [4.3–4.5]	4.2 [4.1–4.2]	**0.003**
LVEF, %	59 [45–60]	56 [50–60]	0.68	58 [50–60]	55 [47–60]	0.72
**Laboratory investigations**						
Fibrinogen, g/L	3.6 [3.2–4.1]	3.6 [3.3–4.1]	0.73	4.1 [3.8–4.3]	3.5 [3.1–3.6]	**0.02**
Creatinine, μmol/L	78.6 ± 13.1	85.4 ± 11.4	0.058	84.5 [80.0–90.0]	89.5 [77.0–97.0]	0.46
CRP, mg/L	2.2 [1.0–4.4]	1.3 [1.0–2.4]	0.44	1.2 [1.0–3.0]	1.4 [1.0–2.3]	0.68
Glucose, mmol/L	5.5 [5.2–6.0]	5.5 [5.1–6.5]	0.93	5.4 [5.2–7.2]	5.6 [4.9–6.0]	0.72
Total cholesterol mmol/L	3.9 [3.4–4.3]	3.6 [2.8–4.8]	0.49	3.6 [2.8–4.5]	3.8 [3.1–5.5]	0.54
LDL cholesterol, mmol/L	2.2 [1.9–2.7]	2.2 [1.6–2.9]	0.82	2.2 [1.5–2.7]	2.2 [1.7–3.2]	0.39
HDL cholesterol, mmol/L	1.2 [1.1–1.5]	1.2 [0.9–1.4]	0.27	1.1 [1.0–1.4]	1.2 [0.9–1.5]	0.82
Triglycerides, mmol/L	1.3 [1.0–1.6]	1.1 [0.8–1.5]	0.17	1.1. [0.9–1.5]	1.0 [0.8–1.5]	0.63

*Note:* Data presented as numbers (percentages), mean ± SD or median and quartiles [Q1–Q3]. *p*‐values of < 0.05 (bold) were considered statistically significant.

Abbreviations: ACE inhibitors, angiotensin converting enzyme inhibitors; AS, aortic stenosis; AVA, aortic valve area; BMI, body mass index; CRP, C‐reactive protein; DOACs, direct oral anticoagulants; LVEF, left ventricular ejection fraction; PG_max_, maximal transvalvular pressure gradient; PG_mean_, mean transvalvular pressure gradient; *V*_max_, peak transvalvular velocity.

### 3.2 In Loco Expression of Calcification Markers

**FIGURE 1:**
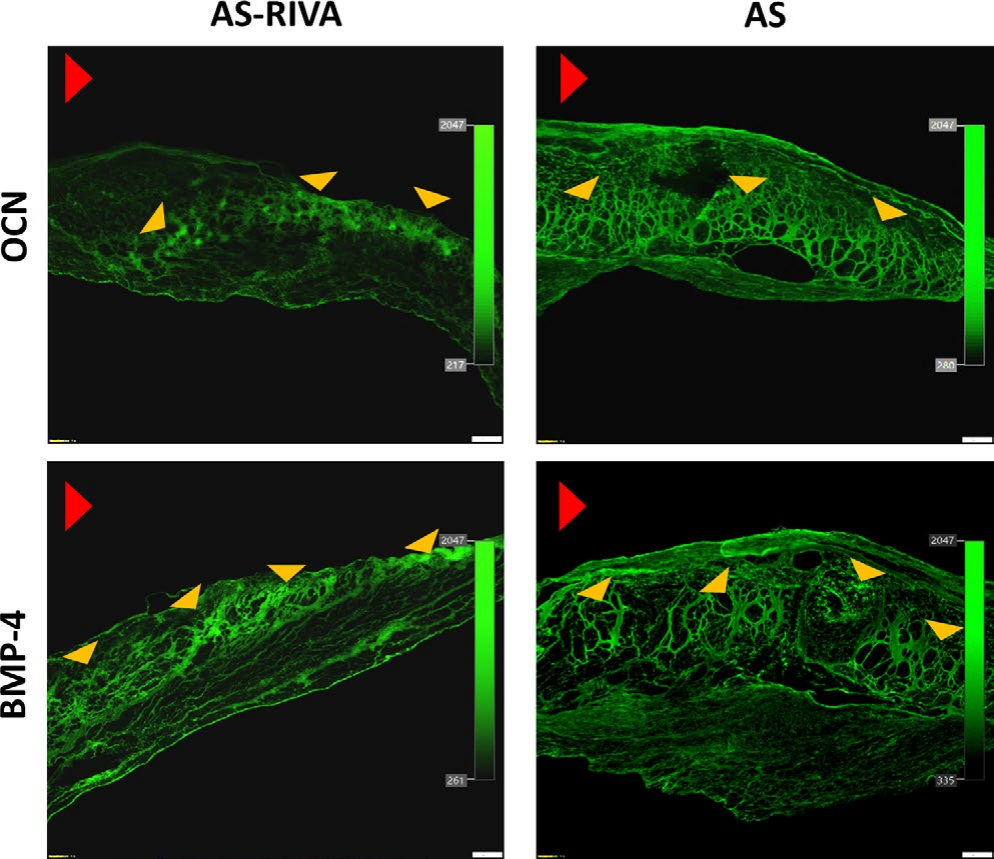
Valvular expression of OCN and BMP‐4. Representative microphotographs show valvular expression of OCN and BMP‐4 within stenotic leaflets in AS patients on rivaroxaban (AS‐RIVA; left panel) and in patients with isolated AS (AS; right panel). Red arrowhead indicates aortic side of the leaflet; yellow arrowheads indicate the immunopositive areas. Scale bar 200 μm, original magnification 4×. BMP‐4, bone morphogenetic protein 4; OCN, osteocalcin.

Immunofluorescence staining of the valve leaflets revealed the presence of BMP‐4 and osteocalcin within valves from patients treated and untreated with DOACs. The average immunopositive area for BMP‐4 in the DOAC group was 26% ± 8.7%, while in the non‐DOAC group, it was 31% ± 9.8% (Figure 1), with no difference between the groups (*p* > 0.05). In contrast, osteocalcin expression was markedly reduced by 39.6% in DOAC‐treated patients (16.3% ± 4.8%) compared to the remainder (27 ± 6.3; *p* < 0.0001) (Figure 1), representing a novel in loco observation.

**FIGURE 2:**
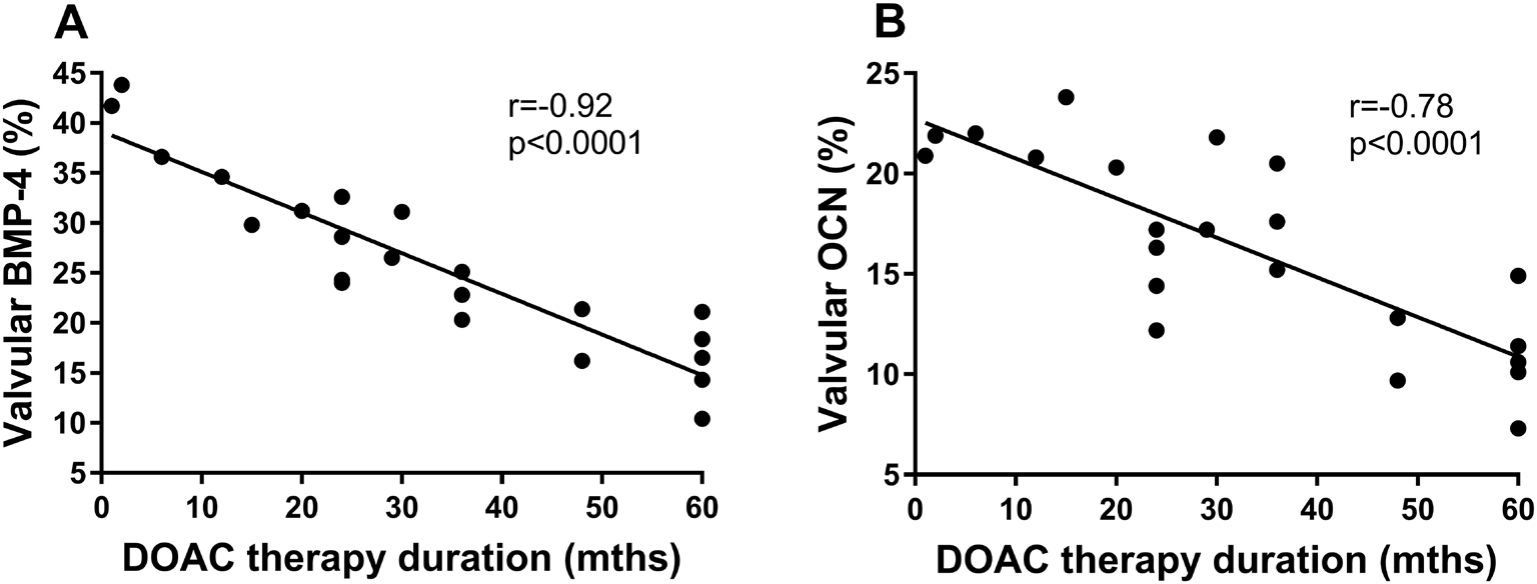
Associations between DOAC treatment time and valvular BMP‐4 or OCN expression. The scatterplots show negative correlations between DOAC therapy duration and valvular expression of (A) BMP‐4 and (B) OCN. DOAC, direct oral anticoagulants. Abbreviations as in Figure 1.

Interestingly, the median thickness of valve leaflets was 35.2% lower in the DOAC group compared to the non‐DOAC group (1119 [788–1135] μm vs. 1727 [1400–1810] μm, *p* < 0.0001). In patients taking DOACs, a trend was observed toward a correlation between BMP‐4 and the AVA (*r* = −0.3, *p* = 0.17). No other correlations were observed between calcification markers and the remaining echocardiographic parameters (all *p* > 0.05). However, the expression of both BMP‐4 and osteocalcin showed a strong negative correlation with the duration of DOAC use (Figure 2), extending our previous observations to additional calcification‐related proteins.

### 3.3 ELISAs Tests

**FIGURE 3:**
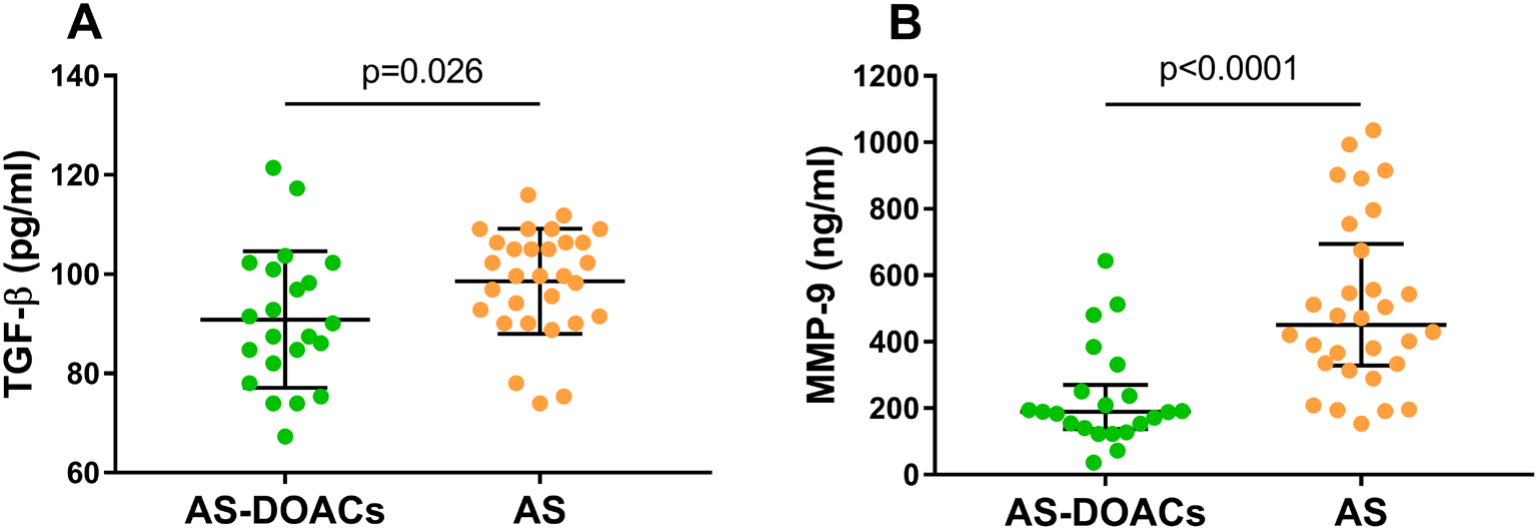
Serum levels of TGF‐β and MMP‐9 in patients with AS. The dot plots show levels of (A) TGF‐β and (B) MMP‐9 in AS patients on DOACs (AS‐DOACs; green dots) and in patients with isolated AS (AS; orange dots). Data presented as mean ± SD for TGF‐β and median and quartiles [Q1‐Q3] for MMP‐9. MMP‐9, matrix metalloproteinase 9; TGF‐β, transforming growth factor‐β.

**FIGURE 4:**
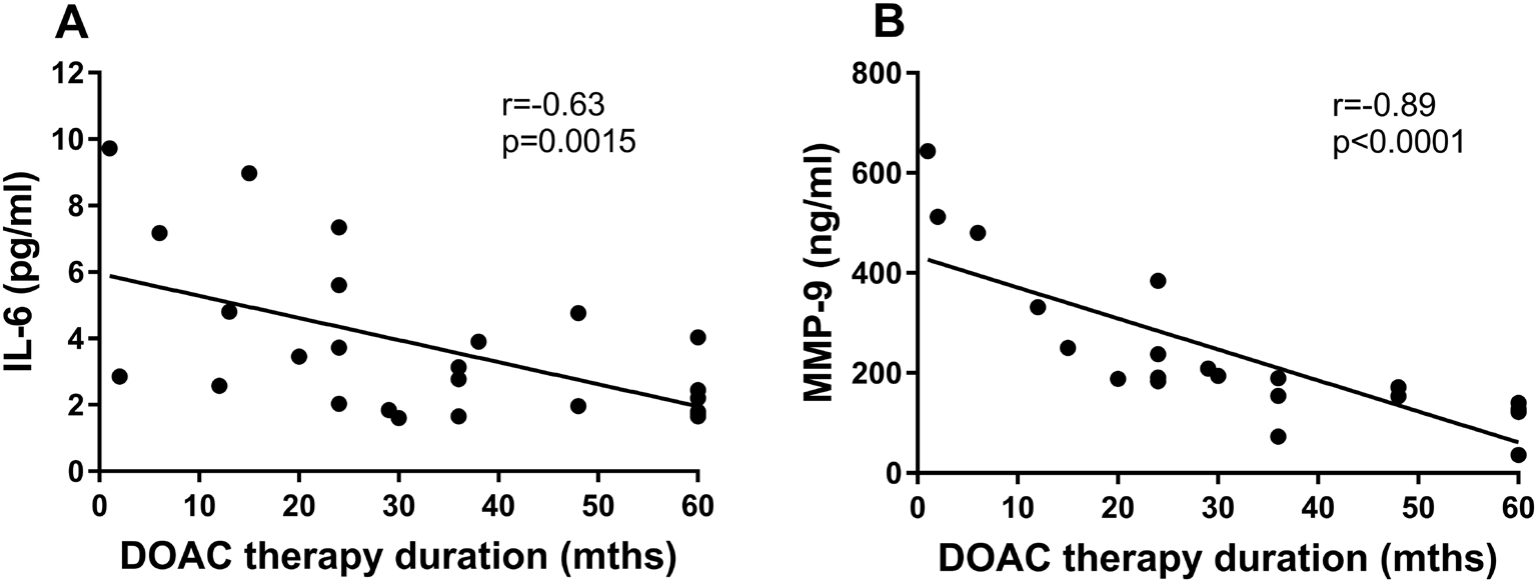
Associations between DOAC treatment time and levels of IL‐6 or MMP‐9. The scatterplots show negative correlations between DOAC therapy duration and serum levels of (A) IL‐6 and (B) MMP‐9. IL‐6, interleukin 6. Abbreviations as in Figures 2 and 3.

No difference was observed in plasma concentrations of TF, a key factor in the activation of the extrinsic coagulation pathway, between the group of patients taking and those not taking DOACs (66 [54.8–70.7] pg/mL vs. 58 [51.2–69.4] pg/mL, respectively; *p* = 0.21). A similar finding was noted for the inflammatory marker hsIL‐6 (2.7 [1.8–4.8] pg/mL in DOAC‐treated patients vs. 4.0 [2.5–5.6] pg/mL in non‐DOAC‐treated subjects, *p* = 0.092). Interestingly, serum levels of TGF‐β (a marker of inflammation and osteoblastic transformation) and MMP‐9 (a marker of tissue remodelling) were significantly lower in the DOAC group compared to the non‐DOAC group (90.8 ± 13.8 pg/mL vs. 98.6 ± 10.6 pg/mL for TGF‐β and 189 [140–250] ng/mL vs. 451 [334–674] ng/mL for MMP‐9, respectively) (Figure 3). In addition, an analysis considering the DOAC treatment time revealed negative correlations between the duration of anticoagulant use and the serum hsIL‐6 and MMP‐9 levels (Figure 4). We also observed that the duration of DOAC therapy negatively correlated with the fibrinogen concentration (*r* = −0.63, *p* = 0.0015). No correlations were observed between levels of studied markers and the echocardiographic parameters in both groups.

### 3.4 In Vitro Effects of Rivaroxaban

#### 3.4.1 NF‐κB Expression

**FIGURE 5:**
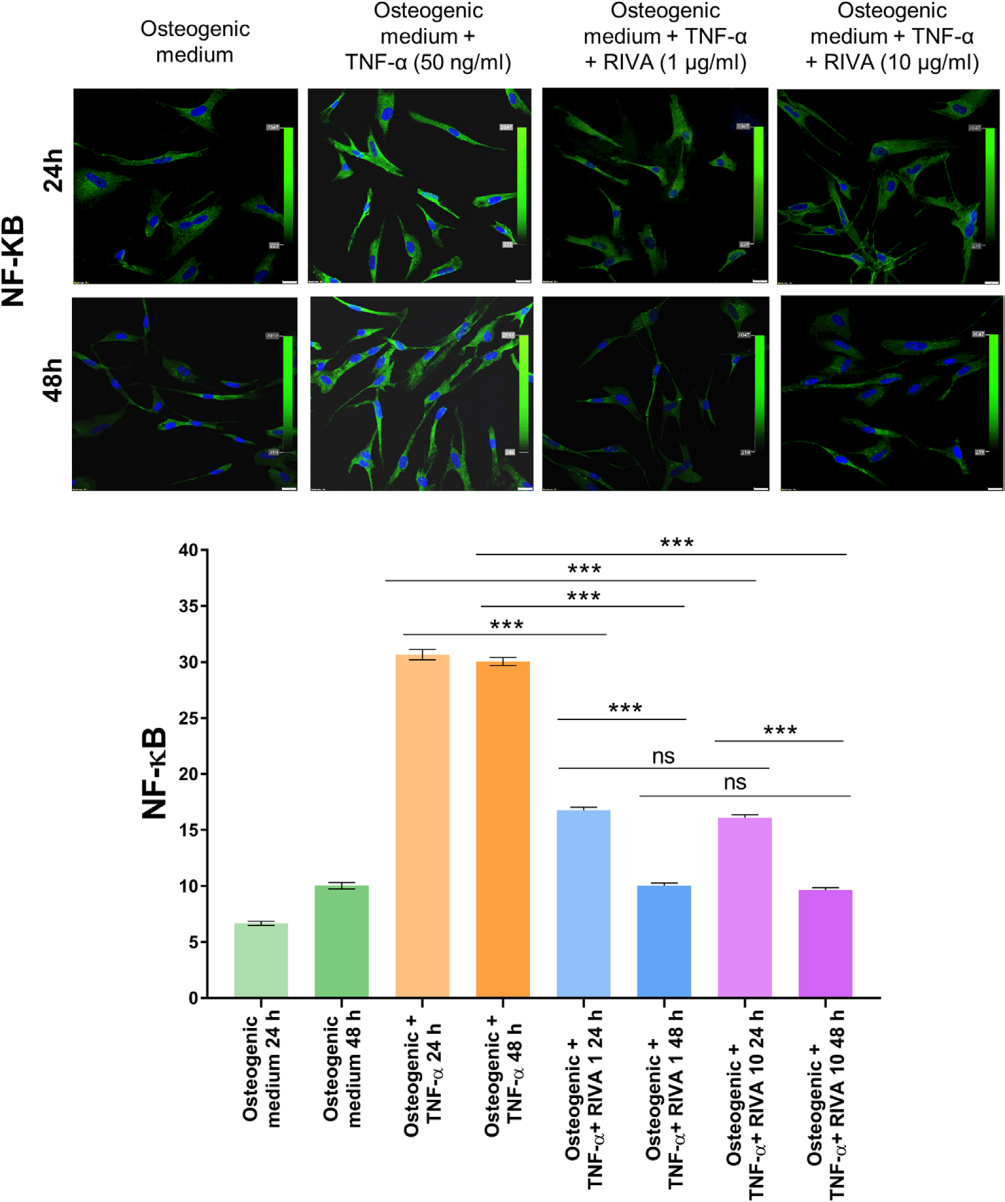
NF‐κB expression in VICs under different culture conditions and the effect of rivaroxaban. (top panel) Representative immunofluorescence images showing NF‐κB expression (green) in VICs cultured under different conditions for 24 and 48 h. Cell nuclei are stained with DAPI (blue). Scale bar = 20 μm, original magnification 40×. (bottom panel) Quantitative analysis of NF‐κB fluorescence intensity in VICs after 24 and 48 h of incubation. Cells were cultured in osteogenic medium, osteogenic medium with TNF‐α or with TNF‐α plus rivaroxaban at 1 or 10 μg/mL. Data are shown as mean ± SD. *p < 0.01, **p < 0.001, ***p < 0.0001, ns, not significant (*p* > 0.05). ANOVA with post hoc tests was used for statistical analysis. Experiments were repeated three times using VICs from different patients; all samples were analysed in triplicate. NF‐κB, nuclear factor‐κB; TNF‐α, tumour necrosis factor alpha; VICs, valve interstitial cells.

TNF‐α stimulation enhanced NF‐κB expression in VICs, showing a 359% increase after 24 h compared to control VICs cultured in osteogenic medium (6.67 ± 0.2 vs. 30.65 ± 0.47 FI; Figure 5). Treatment with rivaroxaban at either 1 μg/mL (R1) or 10 μg/mL (R10) for 24 h reduced NF‐κB expression by 45.3% and 47.5%, respectively, compared to TNF‐α‐stimulated cells, with no significant dose‐dependent difference observed (30.65 ± 0.47 vs. 16.78 ± 0.28 FI and 30.65 ± 0.47 vs. 16.10 ± 0.26 FI; Figure 5).

After 48 h of treatment, both rivaroxaban doses led to a more pronounced suppression of NF‐κB expression, showing an approximate 67% reduction relative to TNF‐α stimulation (R1, 10.03 ± 0.23 vs. 30.05 ± 0.36 FI and R10, 9.64 ± 0.21 vs. 30.05 ± 0.36 FI; Figure 5).

When comparing the effect of rivaroxaban on NF‐κB expression at the two time points, after 48 h, NF‐κB expression was approximately 40% lower compared to 24 h of incubation, irrespective of dose (R1: 10.03 ± 0.23 vs. 16.78 ± 0.28 FI and R10: 9.6 ± 0.21 vs. 16.10 ± 0.26 FI; Figure 5), which is a novel in vitro observation.

#### 3.4.2 BMP‐4 Expression

**FIGURE 6:**
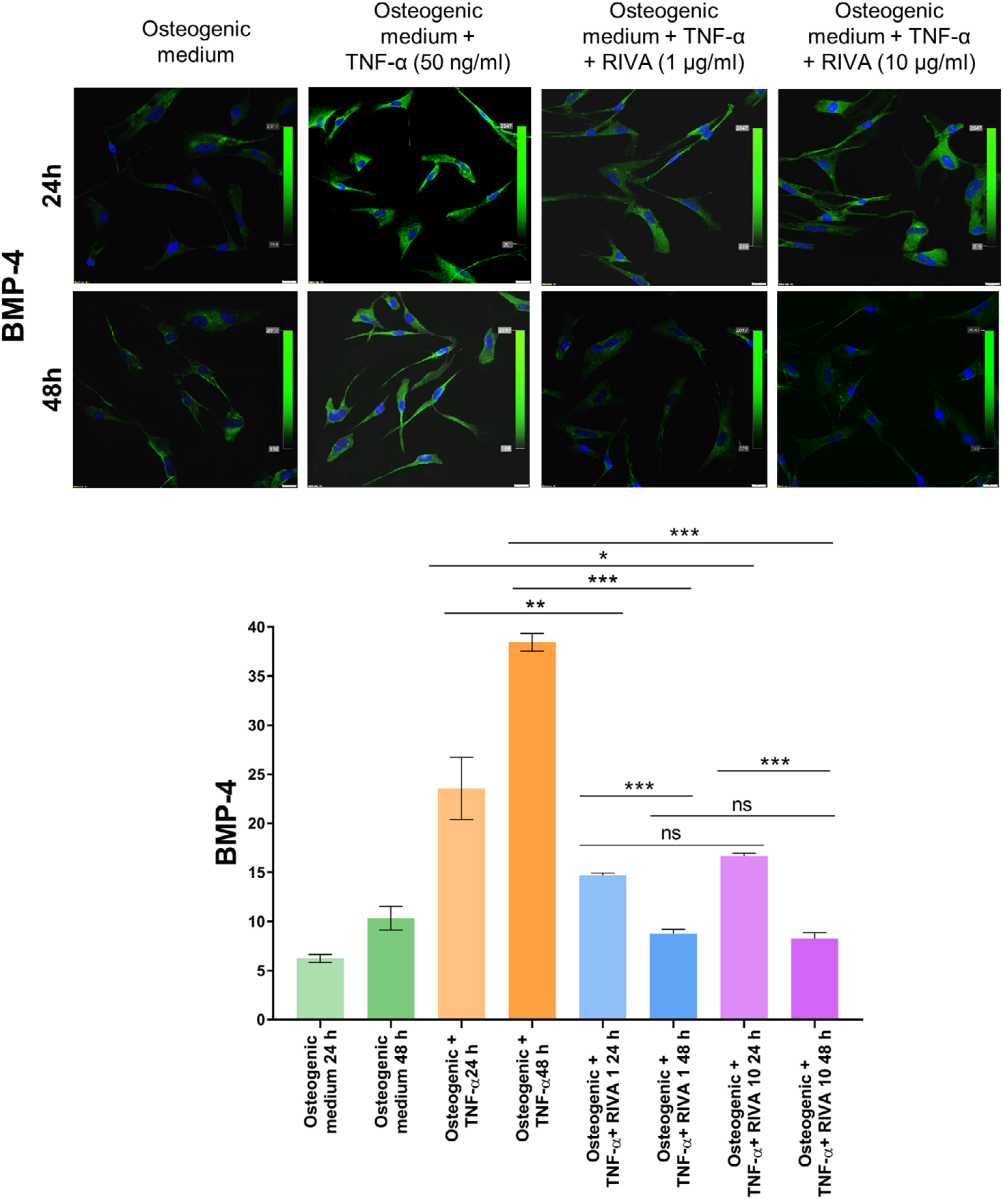
BMP‐4 expression in VICs under different culture conditions and the effect of rivaroxaban. (top panel) Representative immunofluorescence images showing BMP‐4 expression (green) in VICs cultured for 24 and 48 h under various conditions. Cell nuclei are counterstained with DAPI (blue). Scale bar = 20 μm, original magnification 40×. (bottom panel) Quantitative analysis of BMP‐4 fluorescence intensity in VICs after 24 h and 48 h of incubation under the same conditions as in Figure 5: Osteogenic medium, TNF‐α, TNF‐α plus rivaroxaban at 1 μg/mL or 10 μg/mL. Data are presented as mean ± SD. *p < 0.01, **p < 0.001, ***p < 0.0001, ns, not significant (*p* > 0.05). Statistical analysis was performed using ANOVA with appropriate post hoc tests. All experiments were conducted in triplicate using VICs from three independent donors.

BMP‐4 expression increased 277% following TNF‐α stimulation for 24 h compared to control VICs (23.55 ± 3.18 FI vs. 6.24 ± 0.41 FI; Figure 6). After 24 h of rivaroxaban treatment, this increase was attenuated, with BMP‐4 expression decreasing by 37.5% for R1 and 29.3% for R10 compared to TNF‐α‐only treated cultures (14.71 ± 0.22 FI vs. 23.55 ± 3.18 FI and 16.66 ± 0.29 FI vs. 23.55 ± 3.18 FI; Figure 6). At the 48‐h point, BMP‐4 expression was further suppressed, with reductions of 77.2% for R1 and 78.5% for R10 compared to TNF‐α stimulation (8.75 ± 0.45 FI vs. 38.45 ± 0.9 FI and 8.25 ± 0.63 FI vs. 38.45 ± 0.9 FI; Figure 6).

Additionally, BMP‐4 expression after 48 h treatment was approximately 40% lower for R1 and 50% lower for R10 compared to their respective 24 h treatments (R1: 8.75 ± 0.45 FI vs. 14.71 ± 0.22 FI and R10: 8.25 ± 0.63 FI vs. 16.66 ± 0.29 FI; Figure 6), again reflecting a time‐dependent, but not dose‐dependent pattern as a new mechanistic insight.

## 4 Discussion

This study provides the first evidence that rivaroxaban is associated with a reduction in VICs inflammation and calcification in a time‐dependent manner, regardless of the dose. Unlike previous studies, which reported reduced levels of calcification and inflammatory markers in conditioned media, our work demonstrates intracellular modulation of VIC signalling pathways, specifically decreased NF‐κB and BMP‐4 expression. Moreover, we showed that the duration of DOAC therapy was associated with reduced valvular expression of both BMP‐4 and osteocalcin, thereby extending earlier observations limited to osteopontin and NF‐κB. Additionally, subjects treated with DOACs were characterised by lower valvular leaflet thickness.

It is also important to note that in our study, patients on DOACs exhibited less severe AS, as reflected by lower *V*_max_ and reduced transvalvular pressure gradients, particularly among patients treated long‐term (≥ 29 months) with no difference in AVA. Although AVA was similar, transvalvular gradients may still vary, as they are flow‐dependent and influenced by factors such as left ventricular function. In contrast, the analysis of valvular calcification markers' expression revealed only a trend toward a negative correlation with AVA in the DOAC group but not with other echocardiographic parameters. This discrepancy could be related to a small sample size or the study population consisting of patients with severe AS, characterised by heavy valve calcifications and high BMP expression, where such differences might be more evident in earlier stages of the disease. Additionally, osteocalcin expression was highly variable and was assessed through tissue fluorescence intensity, making direct comparison with BMP‐4 expression challenging.

### 4.1 Calcification

While the interplay of various mechanisms involved in the valvular leaflet calcification is broadly studied, the impact of DOACs on this process is yet to be explained (Figure S1). Our previous study found that DOAC‐treated VICs expressed reduced levels of calcification‐related proteins (osteocalcin and osteopontin) detected in the conditioned medium, without addressing the intracellular responses of VICs [15]. The current study for the first time showed a time‐dependent, but not dose‐dependent, reduction in intracellular NF‐κB and BMP‐4 expression following rivaroxaban treatment, based on about 50% lower expression of both proteins after 48 h of rivaroxaban treatment compared to 24 h, whereas no differences were observed between rivaroxaban doses at the same time point. These observations may represent a new mechanistic insight into its action on VICs. Moreover, a similar effect of rivaroxaban was observed in loco, namely, an inverse correlation between rivaroxaban therapy duration and the expression of osteocalcin and BMP‐4 was found. Importantly, our very recent report showed that the volume of valvular calcium deposits measured using micro‐CT correlated with the duration of rivaroxaban therapy, underscoring a potential modulatory effect of rivaroxaban on the progression of valvular calcification in this clinical context [21]. Interestingly, in the animal study performed by Rattazzi et al. [22] mice treated with rivaroxaban had lower calcium accumulation within the aortic valve in comparison with warfarin; however, no differences were found between the rivaroxaban and the control group. The discrepancy might be explained by the difference in the duration of DOAC therapy. Mice were treated with rivaroxaban for 8 weeks [22] and the median duration of DOAC therapy among our patients was 29.5 months. Another study performed on patients with chronic kidney disease and atrial fibrillation showed that rivaroxaban was associated with a significant reduction of both mitral and aortic valve calcification assessed by echocardiography compared to the warfarin‐treated group after a mean 16‐month follow‐up [23]. Moreover, Tastet et al. [24] found that in patients with at least mild AS, the median annual increase in *V*_max_ and aortic valve calcification score was about three times greater in the warfarin group compared to the DOAC group. Although this study was designed to assess whether DOACs, unlike warfarin, may inhibit valve calcification, it did not provide evidence of whether DOACs can inhibit this process. However, these findings, complemented with our observations, may suggest that DOACs exert a beneficial therapeutic effect in AS patients through their pleiotropic actions, especially since a significantly lower valve leaflet thickness (by 35.2%) was observed in patients taking DOACs. In aortic stenosis, leaflet thickening is closely linked to inflammation‐driven calcification. The reduced leaflet thickness may reflect rivaroxaban anti‐inflammatory and anti‐fibrotic effects, potentially limiting extracellular matrix remodelling, fibrosis and osteogenic signalling. Although baseline AS severity may have contributed, our in vitro data show that rivaroxaban downregulates NF‐κB and BMP‐4 expression in VICs, supporting its role in modulating leaflet thickening pathways. This is further supported by Hara et al. [25], who found that rivaroxaban attenuates atherosclerotic plaque progression in ApoE‐deficient mice. Given shared mechanisms between atherosclerosis and AS, including inflammation, lipid accumulation and calcification, these findings may suggest DOAC‐dependent suppression of valvular inflammation and fibrotic remodelling, resulting in reduced leaflet thickness. However, further studies with larger patient cohorts are needed to confirm this hypothesis.

### 4.2 Inflammation and Coagulation

In DOAC‐treated patients, serum TGF‐β levels were found to be lower compared with patients not taking DOACs, whereas no such differences were observed for CRP and hsIL‐6. This may be explained by the AS‐associated inflammation being localised primarily within the valve and much less pronounced peripherally [26], highlighting the limitation of analysing systemic markers alone. However, we observed a negative correlation between the duration of DOAC therapy and hsIL‐6 serum levels in AS patients. Therefore, demonstrating the impact of DOACs on these mechanisms may require more refined analysis of in loco inflammatory markers or studies involving larger patient cohorts using these drugs for extended periods. Importantly, our recent study provided additional in loco evidence for the anti‐inflammatory effects of rivaroxaban, demonstrating decreased NF‐κB expression and lower IL‐6 fluorescence intensity within stenotic leaflets from AS patients receiving rivaroxaban compared with non‐DOAC users [21]. Furthermore, valvular NF‐κB expression correlated inversely with the duration of rivaroxaban therapy and positively with micro‐CT measures of calcification, underscoring its role as a mediator linking inflammatory signalling with structural valve degeneration [21]. These histological observations are consistent with our novel in vitro findings, where rivaroxaban treatment was associated with lower NF‐κB expression in VIC cultures in a time‐dependent but dose‐independent manner, suggesting a cellular mechanism through which DOACs may influence valvular inflammation. Moreover, several studies have investigated the impact of DOACs on inflammatory processes, albeit primarily in animal models and in the context of atherosclerotic vascular disease [25, 27]. Dabigatran was shown to down‐regulate NF‐κB and MMP‐9 expression in murine aortic plaques [27]. Furthermore, DOACs attenuated atherosclerotic plaque development in mice by reducing lipid deposition and macrophage accumulation [25]. Consistently, rivaroxaban was shown to inhibit NF‐κB expression in human atrial myofibroblast cultures [28].

We also observed that the duration of DOAC use was negatively associated with plasma fibrinogen levels. Although several medications, including statins and fibrates, were shown to decrease fibrinogen concentration, to prove such an effect associated with DOAC treatment, a larger study is required [29]. However, in our opinion, it is most plausible that fibrinogen levels decrease as a result of DOAC‐mediated suppression of inflammation.

This may be beneficial for AS patients, as coagulation activation is known to be involved in AS progression and valvular degeneration [11, 20, 30]. Our previous data showed that elevated TF expression was associated with AS severity [11]. In vitro studies on VIC cultures showed a constant expression of TF, which was upregulated when VICs were stimulated with inflammatory cytokines [15]. Exposure of VIC cultures to therapeutic concentrations of rivaroxaban (1 or 10 ng/mL) significantly reduced expression of factor Xa and TF. While plasma TF levels did not differ between DOAC‐treated and untreated patients in our study, this does not exclude the impact of DOACs on TF expression within aortic valves. On the other hand, we found lower serum MMP‐9 levels in the DOAC group compared with non‐DOAC patients. Additionally, we observed a negative correlation between DOAC treatment time and serum MMP‐9 concentration. These results might suggest that DOACs can impact extracellular matrix remodelling equilibrium, possibly limiting valvular remodelling and calcification since extracellular matrix remodelling seems to play a crucial role in aortic valve calcification [15, 31]. Further studies are needed to test this hypothesis.

## 5 Study Limitations

This study has several limitations. The overall sample size was relatively small, particularly in the subgroup of patients taking DOACs, which may reduce statistical power for some analyses. Nevertheless, we enrolled all consecutive AS patients receiving DOACs who were available during the study period. In addition, the duration of DOAC therapy was a crucial factor influencing the analysed parameters. Therefore, future studies should focus on patients taking DOACs for longer than 29 months to better elucidate the long‐term effects of these agents. Second, the clinical assessment included patients treated with various DOACs (rivaroxaban, apixaban, dabigatran), while the in vitro experiments were conducted exclusively with rivaroxaban, which was also the most commonly used agent in our patients. This heterogeneity may limit the generalisability of mechanistic findings to other DOACs, and further studies are needed to determine whether different agents exert comparable effects on valvular calcification. Third, since differences in echocardiographic parameters between DOAC‐treated and untreated patients may partly reflect earlier referral for surgical valve replacement, we made efforts to minimise such bias. We included patients with a diagnosis of AS made at least 5 years prior, excluded those needing emergency surgery, and matched participants for key clinical factors. All patients had isolated AS without coexisting atherosclerosis.

Fourth, the expression of the studied factors in the valves was determined semi‐quantitatively, which may affect the accuracy of the results. Moreover, there is a risk of systematic errors that could have occurred at various stages of conducting experiments and analyses. However, to increase the reliability of the results, valve staining and morphometric measurements were conducted on multiple samples for each patient, with each sample containing three sections. The obtained results are only preliminary observations, and the presented associations do not mean the cause–effect relationship; therefore, more detailed studies are needed. Additionally, only selected markers of calcification, inflammation and coagulation activation were examined.

Fifth, we did not include direct assessment of total calcification (e.g., Alizarin Red or von Kossa staining), as such methods often yielded non‐specific results in VICs due to their high osteogenic potential. Instead, we focused on the expression of selected calcification markers. Additionally, as a hypothesis‐generating study, the mechanistic analysis was limited; broader profiling of signalling pathways (e.g., Runx2, alkaline phosphatase, Smads, Wnt/β‐catenin) was not performed. Future investigations are required to reveal the impact of DOACs on these pathways. It would also be important to enrich the analysis with quantitative protein assessment methods, such as proteomic analyses, which could provide more precise data. In addition, genomic‐level analysis (e.g., qPCR) could significantly enhance our understanding of the impact of DOACs on AS progression.

Finally, although the aortic valve is composed of both VICs and VECs, the present study focused exclusively on VICs. To our knowledge, no studies have examined the effects of DOACs on VECs. However, pleiotropic actions of DOACs in vitro on endothelial cell cultures have been reported [32]. Future studies incorporating VEC analyses will be essential to provide a more comprehensive understanding of the cellular mechanisms underlying valve pathobiology.

## 6 Conclusion

This study showed that rivaroxaban exerts anti‐inflammatory and anti‐calcific effects on VICs in a time‐dependent but not dose‐independent manner. In patients with severe AS, long‐term DOAC therapy was further associated with lower expression of calcification markers within stenotic leaflets, as well as lower levels of circulating MMP‐9 and IL‐6. These findings suggest that long‐lasting rivaroxaban treatment may slow the rate of AS progression. While our results highlight the therapeutic potential of DOACs in AS, further prospective studies are warranted to validate these observations and to establish their clinical relevance, particularly in the earlier stages of disease.

## Author Contributions

**Magdalena Kopytek:** data curation (lead), formal analysis (lead), investigation (lead), methodology (lead), writing – original draft (lead). **Marcin Zuwała:** investigation (supporting), writing – original draft (supporting). **Radosław Chudy:** investigation (supporting), writing – original draft (supporting). **Weronika Włóczyk:** investigation (supporting). **Michał Ząbczyk:** formal analysis (supporting), investigation (supporting), writing – original draft (supporting). **Jacek Piątek:** investigation (supporting). **Anetta Undas:** conceptualization (supporting), validation (supporting). **Joanna Natorska:** conceptualization (lead), funding acquisition (lead), investigation (supporting), project administration (lead), supervision (lead), validation (lead).

## Conflicts of Interest

The authors declare no conflicts of interest.

## Supporting information


**Figure S1:** jcmm70927‐sup‐0001‐FigureS1.jpeg.

**Appendix S1:** jcmm70927‐sup‐0002‐AppendixS1.docx.

## Data Availability

The data that support the findings of this study are available from the corresponding author upon reasonable request.
