# Supplementary figures and images for: Duration‐Dependent Effects of Rivaroxaban on Inflammation and Valve Calcification in Aortic Stenosis: Clinical and In Vitro Insights
Source: J Cell Mol Med. 2025 Oct 31;29(21):e70927. doi: 10.1111/jcmm.70927 (PMC12578597; doi:10.1111/jcmm.70927)

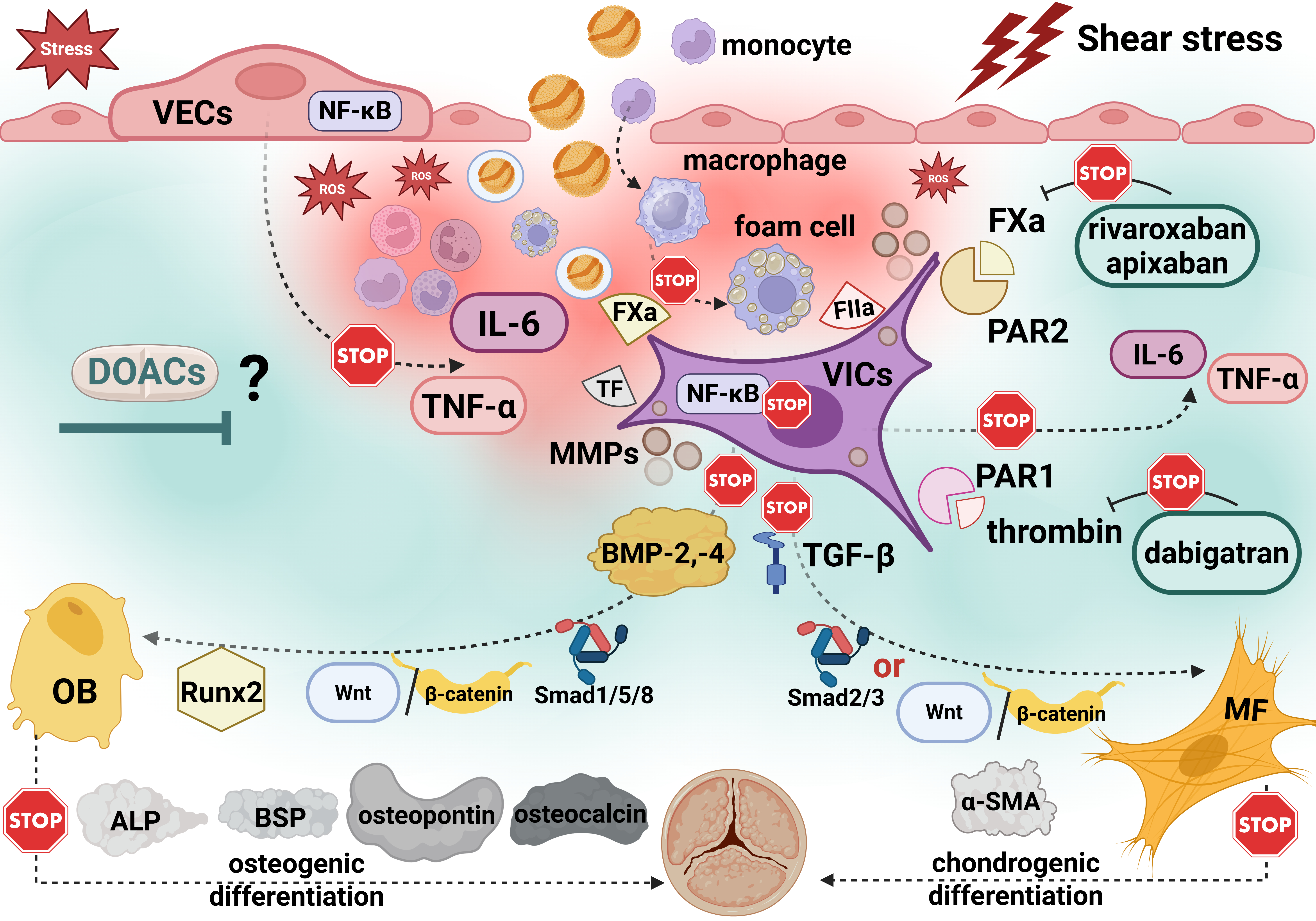

Supplement: Supplementary file 1 — Figure S1: jcmm70927‐sup‐0001‐FigureS1.jpeg. [file JCMM-29-e70927-s001.jpeg]
