# Supplementary material for: Duration‐Dependent Effects of Rivaroxaban on Inflammation and Valve Calcification in Aortic Stenosis: Clinical and In Vitro Insights
Source: J Cell Mol Med. 2025 Oct 31;29(21):e70927. doi: 10.1111/jcmm.70927 (PMC12578597; doi:10.1111/jcmm.70927)
Supplement: Supplementary file 2 — Appendix S1: jcmm70927‐sup‐0002‐AppendixS1.docx. [file JCMM-29-e70927-s002.docx]

**
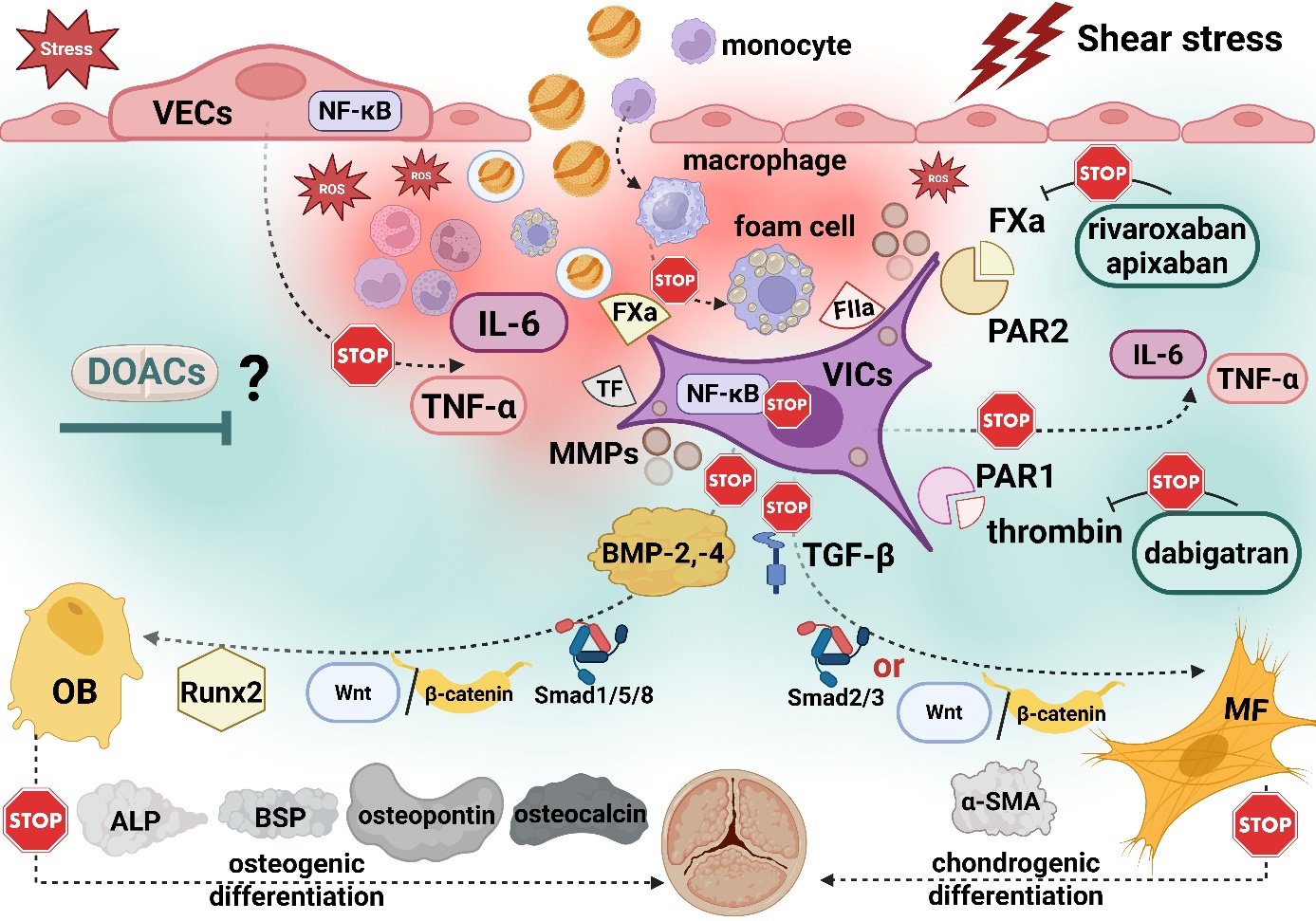
**

**Abbreviations:**

ALP – alkaline phosphatase, α-SMA – α-smooth muscle actin, BMP-2/4 – bone morphogenetic protein-2/4, BSP – bone sialoprotein, DOACs – direct oral anticoagulants, FII – thrombin, FXa – active factor X, IL-6 – interleukin-6, MF – myofibroblast, MMPs – matrix metalloproteinases, NF-κB – nuclear factor-κB, OB – osteoblasts, PAR1/2 – protease-activated receptor 1/2, ROS – reactive oxygen species, Runx2 – Runt-related transcription factor 2, TF – tissue factor, TGF-β – transforming growth factor-β, TNF-α – tumor necrosis factor-α, VECs – vascular endothelial cells, VICs – valve interstitial cells.

**Figure S1. Effect of DOACs on valve calcification and inflammation in aortic stenosis.** The initial stage of aortic valve calcification is endothelial damage caused by high shear stress, which arises from altered hemodynamics and turbulent blood flow across the narrowed aortic valve. Valve endothelial cells (VECs) normally act as a protective barrier, limiting lipid accumulation and inflammatory cell infiltration. Valve interstitial cells (VICs), residing in all valvular layers, are the predominant cell type within the valve and play a central role in the pathogenesis of aortic stenosis (AS) through extracellular matrix remodeling, fibrosis, and osteogenic differentiation.

Endothelial dysfunction, induced by shear and oxidative stress, facilitates the recruitment of circulating monocytes and the accumulation of lipoproteins. Elevated reactive oxygen species (ROS) promote lipoprotein oxidation, followed by their phagocytosis by infiltrating macrophages, leading to foam cell formation and initiation of local inflammation. In affected valves, activation of nuclear factor κB (NF-κB), a master regulator of inflammatory responses, enhances the expression of pro-inflammatory and coagulation-related mediators, which may be inhibited by direct oral anticoagulants (DOACs). NF-κB also governs key steps in valvular calcification. Its activation can be induced by tumor necrosis factor-α (TNF-α) or transforming growth factor-β (TGF-β), which increase interleukin-6 (IL-6) production and amplify local inflammation, thereby promoting calcification through the induction of osteoblast-associated genes. The transformation of VICs into myofibroblasts (MF) - chondrogenic differentiation and osteoblast-like fibroblasts (OB) phenotype - osteogenic differentiation is regulated by cytokines. Concurrently, bone morphogenetic proteins (BMPs) and TGF-β initiate osteogenic and fibrotic pathways in VICs through Smad and Wnt/β-catenin signaling, upregulating Runt-related transcription factor 2 (Runx2), which further drive osteoblast-like differentiation and the expression of calcification-related proteins. Attenuation of inflammatory signaling may downregulate the expression of osteogenic proteins in VICs and inhibit fibrotic and calcific remodeling of the valve. Being at least in part under NF-κB control, calcification pathways can also be inhibited by DOACs.

Proteolytic activation of protease-activated receptors (PARs) by either thrombin (FIIa) or activated factor X (FXa) triggers downstream signaling in both VICs and VECs, linking coagulation with inflammatory and osteogenic pathways. FXa activates PAR-1 and PAR-2, while thrombin primarily activates PAR-1. In VECs, PAR activation induces calcium influx and production of inflammatory mediators such interleukins, promoting endothelial activation and immune cell recruitment. In VICs, PAR1 and PAR2 signaling drives proliferation, migration, adhesion, apoptosis, inflammation, and pro-calcific signaling. Given the multifactorial nature of AS progression, it may be hypothesized that DOACs, by inhibiting FXa and/or thrombin, interfere with the early activation of PAR-mediated signaling and reduce NF-κB-driven inflammation. However, many of the DOAC-driven mechanisms in AS remain largely unknown, and current understanding allows us only to hypothesize their potential pleiotropic effects on key pathological processes.
